# Mayer-Rokitansky-Küster-Hauser (MRKH) syndrome

**DOI:** 10.1186/1750-1172-2-13

**Published:** 2007-03-14

**Authors:** Karine Morcel, Laure Camborieux, Programme de Recherches sur les Aplasies Müllériennes (PRAM), Daniel Guerrier

**Affiliations:** 1 CNRS UMR 6061, Institut de Génétique et Développement de Rennes (IGDR), Université de Rennes 1, IFR140 GFAS, Faculté de Médecine, Rennes, France; 2 Département d'Obstétrique, Gynécologie et Médecine de la Reproduction Hôpital Anne de Bretagne, Rennes, France; 3 Association MAIA, Toulouse, France; 4 Programme de Recherches sur les Aplasies Müllériennes (PRAM) – Coordination at: CNRS UMR 6061, Institut de Génétique et Développement de Rennes (IGDR), Université de Rennes 1, IFR140 GFAS, Faculté de Médecine, Rennes, France

## Abstract

The Mayer-Rokitansky-Küster-Hauser (MRKH) syndrome is characterized by congenital aplasia of the uterus and the upper part (2/3) of the vagina in women showing normal development of secondary sexual characteristics and a normal 46, XX karyotype. It affects at least 1 out of 4500 women. MRKH may be isolated (type I) but it is more frequently associated with renal, vertebral, and, to a lesser extent, auditory and cardiac defects (MRKH type II or MURCS association). The first sign of MRKH syndrome is a primary amenorrhea in young women presenting otherwise with normal development of secondary sexual characteristics and normal external genitalia, with normal and functional ovaries, and karyotype 46, XX without visible chromosomal anomaly. The phenotypic manifestations of MRKH syndrome overlap with various other syndromes or associations and thus require accurate delineation. For a long time the syndrome has been considered as a sporadic anomaly, but increasing number of familial cases now support the hypothesis of a genetic cause. In familial cases, the syndrome appears to be transmitted as an autosomal dominant trait with incomplete penetrance and variable expressivity. This suggests the involvement of either mutations in a major developmental gene or a limited chromosomal imbalance. However, the etiology of MRKH syndrome still remains unclear. Treatment of vaginal aplasia, which consists in creation of a neovagina, can be offered to allow sexual intercourse. As psychological distress is very important in young women with MRKH, it is essential for the patients and their families to attend counseling before and throughout treatment.

## Disease name and synonyms

Mayer-Rokitansky-Küster-Hauser (MRKH) syndrome. This syndrome is subdivided in two types: type I (isolated) or Rokitansky sequence (OMIM 277000), and type II or MURCS association (MÜllerian duct aplasia, Renal dysplasia and Cervical Somite anomalies) (OMIM 601076). The MRKH syndrome is also referred to as CAUV (Congenital Absence of the Uterus and Vagina), MA (Müllerian Aplasia) or GRES (Genital Renal Ear Syndrome). It would thus be preferable that all entries (MRKH type I and type II, MURCS association, CAUV, MA and GRES) refer to the unique OMIM number 601076.

## Definition and diagnosis criteria

The MRKH syndrome is characterized by congenital aplasia of the uterus and the upper part (2/3) of the vagina in women showing normal development of secondary sexual characteristics and a normal 46, XX karyotype.

Other associated malformations include (type II or MURCS association):

- Renal (unilateral agenesis, ectopia of kidneys or horseshoe kidney)

- Skeletal and, in particular, vertebral (Klippel-Feil anomaly; fused vertebrae, mainly cervical; scoliosis)

- Hearing defects

- More rarely, cardiac and digital anomalies (syndactyly, polydactyly)

Isolated utero-vaginal aplasia is referred to as Rokitansky sequence or to type I (isolated) MRKH syndrome. Incomplete aplasia and/or associated with other malformations, is generally referred to as MURCS association (or type II MRKH syndrome). In this case, the term GRES (Genital Renal Ear Syndrome) can also be used.

## Epidemiology

The incidence of MRKH syndrome has been estimated as 1 in 4500 female births [1-3]. The majority of cases appears to be sporadic [4], however family cases have also been described [1,5-7]. The mode of inheritance seems to be autosomal dominant with an incomplete degree of penetrance and variable expressivity [1,8,9], suggesting that the prevalence of the syndrome may probably be underestimated. Type I (isolated) MRKH is less frequent than MURCS association [10].

## Clinical description

### Principle features of MRKH syndrome

The first clinical signal is generally a primary amenorrhea in patients presenting with a normal female phenotype, normal 46, XX karyotype [11-14], and normal and functioning ovaries with no sign of androgen excess [15,16]. External examination reveals completed puberty with normal secondary female sexual characteristics (pubic hair and breast development are Tanner stage 5) and normal external genitalia. At the same time, the vagina is reduced to a more or less deep (2–7 cm) vaginal dimple.

Anatomic examination is however necessary to diagnose an MRKH syndrome of either type. Complete uterus aplasia in the presence of two rudimentary horns linked by a peritoneal fold and normal Fallopian tubes correspond to isolated or MRKH type I syndrome [17]. Type II MRKH is characterized by uterine symmetric or asymmetric hypoplasia, accompanied by aplasia of one of the two horns or by a size difference between the two horn rudiments, coupled with tubar malformations such as hypoplasia or aplasia of one or the two tubes [18].

Other malformations are often associated with MRKH type II syndrome and involve the upper urinary tract, the skeleton and the otologic sphere; heart malformations are more rarely reported. In this case, the acronym MURCS is generally used instead [13,19]. Cases of polycystic ovaries [20-22] and ovarian tumors [23-25] have been described in women presenting otherwise with normal 46, XX karyotypes. Moreover, aplasia or absence of Müllerian derivatives suggestive of MRKH syndrome have been described in cases of gonadal dysgenesis [26,27] or agenesis [28,29] in XY or X0 patients presenting with female phenotypes. At present, these types of ovarian pathologies are not considered to be part of the MRKH or MURCS clinical spectrum, since no single group of patients showing a random association between any of these pathologies and utero-vaginal aplasia has been reported so far. However, such studies should be undertaken on large cohorts of women with MRKH, to confirm this assumption.

### Associated malformations in MRKH syndrome type II (MURCS association)

#### Associated upper urinary tract malformations

Altogether, associated upper urinary tract malformations are found in about 40% of cases with MRKH syndrome [18]. Mainly, they include unilateral renal agenesis (23–28%), ectopia of one or both kidneys (17%), renal hypoplasia (4%), horseshoe kidney and hydronephrosis [30,31]. Moreover, a case of bilateral renal agenesis (Potter sequence) associated with absence of uterus and oviducts has been reported in a medically aborted fetus [32], reinforcing the idea that Müllerian aplasia, the principle feature of MRKH syndrome, could be an extra manifestation of hereditary renal adysplasia (HRA) [8] in some cases. At present, we are investigating a family where this type of association has been found: the proband is a 46, XX fetus with no visible chromosomal anomaly; the father and his first daughter (now 5 years old) presented with isolated unilateral renal agenesis. The father's cousin, in addition, showed hemi-uterus, a feature already described in HRA [33,34]. Taking this into account, renal adysplasia seems to be either the prime characteristic of HRA where Müllerian malformations of various types are sometimes encountered or a secondary manifestation of MRKH syndrome. Although similar, these syndromes can probably be distinguished from each other when family histories are available: HRA is transmitted as a strict autosomal dominant trait [35,36], whereas MRKH shows incomplete penetrance coupled with a highly variable expressivity when described in relatives [4,9,37,38]. It is therefore noteworthy that renal evaluation is not only required when diagnosing MRKH syndrome, but is also fully justified in probands relatives.

#### Associated skeletal abnormalities

These anomalies mainly involve the spine (30 to 40%) [18,30,39] and, less frequently, the face and the limb extremities. Rachidial malformations encountered in MURCS association are scoliosis (20%) [30], isolated vertebral anomalies (asymmetric, fused or wedged vertebrae), Klippel-Feil association (fusion of at least two cervical segments, short neck, low hair line, restriction of neck motion) [40] and/or Sprengel's deformity [41], rib malformation or agenesis, and spina bifida [39]. Face and limb malformations are mainly brachymesophalangy [42], ectrodactyly [43], duplicated thumb [44], absent radius [45], atrio-digital dysplasia (Holt-Oram like syndrome) [46,47] and facial asymmetry [48-50].

#### Associated hearing impairment

Auditory defects or deafness are associated with 10 to 25% of MURCS patients [41,51,52]; they often concern conductive deafness due to middle ear malformations, such as stapedial ankylosis [41], or sensorineural defects of varying severity [52]. Patients with hearing loss associated with adysplasia of the auditory meatus and/or malformed ears have also been reported [48,53].

#### Associated heart malformations

The association of MRKH with heart malformations is less common. All reports involved lethal or severe cardiac defects evocating Holt-Oram or velocardiofacial-like syndromes requiring surgery when possible. Such reported malformations were aorto-pulmonary window [47], atrial septal defect [46] and conotruncal defects such as pulmonary valvular stenosis [54] or Tetralogy of Fallot [55].

## Etiology

The MRKH syndrome was initially considered to be of sporadic occurrence, suggesting the involvement of non-genetic or environmental factors [56] such as gestational diabetes [57] or thalidomide-like teratogens [1,13,30,58]. However, studies analyzing available pregnancy histories failed to identify any association with drug use, illness, or exposure to known teratogens [57,59-61]. Another explanation of the sporadic occurrence of the syndrome was the hypothesizes of a polygenic/multifactorial inheritance [4,38,56,62], characterized by a low recurrence risk for first-degree relatives. The most plausible explanation actually relies on the description of significant and increasing number of familial aggregates based on accurate delineation of the syndrome in the probands as well as in their relatives. Indeed, utero-vaginal aplasia is often found associated with other malformations, mainly renal and skeletal, these two latter being sometimes observed in combination with the first and interestingly, occurring in more distant relatives as well as mothers of MRKH patients [1,6,8,9,63]. Utero-vaginal aplasia can thus represent only one manifestation of a variably expressed genetic defect. This latter appears to be transmitted as an autosomal dominant trait with incomplete penetrance coupled with variable expressivity of a single mutant gene, as previously hypothesized [1,8,9,64], or of a limited chromosomal imbalance undetectable in standard karyotypes.

The etiology of MRKH syndrome has remained quite unclear until now [64,65], although the spectrum of malformations encountered suggests a developmental field defect [13,19], involving organ systems which are closely related during embryogenesis. More precisely, MRKH syndrome may be attributed to an initial affection of the intermediate mesoderm, consequently leading (by the end of the fourth week of fetal life) to an alteration of the blastema of the cervicothoracic somites and the pronephric ducts [13]. These latter subsequently induce the differentiation of the mesonephroi and then the Wolffian and Müllerian ducts.

The lack of families with informative genetic histories has initially led to a candidate gene approach for determination of the underlying etiology of the syndrome based either on association with other genetic diseases or on involvement during embryogenesis. As a result, the genetic association of MRKH with galactosemia [66] or with cystic fibrosis [67] was analyzed, but neither the gene for galactose-1-phosphate uridyl transferase (*GALT*) [68] nor the gene encoding the cystic fibrosis transmembrane regulator (CFTR) chloride channel [67] showed any mutation or polymorphism associated with the disorder. Aberrant expression of anti-Müllerian hormone (AMH) or its receptor, both involved in Müllerian duct regression [69] was hypothesized as a cause of MRKH syndrome [2,70]; however, this theory was later discounted as a result of contradictory findings from a study of 32 patients [71]. Moreover, incomplete aplasia of Müllerian structures is often observed in MRKH syndrome, showing that Müllerian differentiation does take place but is incomplete.

Genes with a broad spectrum of activity during early development (such as *WT1 *[72], *PAX2 *[73], *HOXA7 *to *HOXA13 *[64,74] and *PBX1 *[74]) have also been suggested as candidates, on the basis of phenotypes observed in mutant mice. However, their role in MRKH syndrome has not been subsequently demonstrated. *WNT4 *is another developmental gene, belonging to the *WNT *family of genes that regulate cell and tissue growth and differentiation during embryogenesis [75]: its homozygotic inactivation in the mouse model leads to a total failure of Müllerian duct formation and numerous lethal defects at birth [76]. In addition, *WNT4 *is known to be critical for successful nephrogenesis [77-79]. A loss-of-function mutation in the *WNT4 *gene has been recently described in an 18-year-old woman, in association with absence of Müllerian-derived structures, unilateral renal agenesis, and clinical signs of androgen excess [80]. The congenital malformations observed in this patient suggested an MRKH-like phenotype and were similar to those observed in the *Wnt4*-/-mouse [76], indicating a dominant effect [80]. In this pathological case as well as in the mouse model, it seems that loss-of-function of *WNT4 *which is essential for normal ovarian differentiation [76], has led to a masculinization of the fetal gonads consequently producing androgens. The WNT4 protein is known to repress male-specific genes such as those encoding steroidogenic enzymes CYP17A1 and HSB3B2, which are essential for the synthesis of testosterone [76]. Mutated WNT4 may not be able to suppress the expression of androgen-synthesizing enzymes in ovarian cells, therefore leading to the observed hyperandrogenic phenotype [80,81]. Furthermore, WNT4 appears to be essential for the initial differentiation of the Müllerian ducts [65,76,82]. The dominant-negative mutation of *WNT4 *may then produce two distinct effects, hyperandrogenism and uterine aplasia. The sequencing of the *WNT4 *gene in 19 MRKH patients has confirmed that this gene is not involved in MRKH syndrome [83]. Finally, the very recent report on a second patient bearing another *WNT4 *mutation has led to the conclusion that WNT4 deficiency is responsible for a clinical phenotype distinct from the classic MRKH syndrome [81]. This new syndrome due to *WNT4 *mutations in XX women and characterized by absence of Müllerian ducts derivatives, hyperandrogenism and kidney optional adysplasia [80,81], is close but different from MRKH syndrome; therefore, it should be referred to as a proper name, such as "WNT4 syndrome" or "WNT4 defects" and be consequently recorded under an appropriate OMIM number. This latter could well be 277000 if amended; OMIM 601076 would then be restricted to MRKH type I and II or MURCS.

The *TCF2 *gene (formerly *v-HNF1 *or *HNF-1****β***) was originally found associated with MODY-type diabetes [84] and with diabetes mellitus, renal cysts and other renal developmental disorders [85,86]. Interestingly, genital malformations such as bicornuate uterus [87], uterus didelphys [87] and Müllerian aplasia [88] (OMIM 158330) were occasionally found associated with renal anomalies in some familial aggregates showing mutations within the *TCF2 *gene. Defects of this later gene can thus account for some rare cases of Müllerian malformations, including aplasia, making this gene one of the candidates for MRKH, but restricted to familial cases with renal and/or diabetes history. Finally the hypothesis of polygenic/multifactorial causes for MRKH syndrome has been reinforced by recent findings, in adults, of interstitial and terminal deletions involving chromosomes 22 [89] and 4 [90], respectively. However, the large number of genes included in each of these deletions has not allowed yet to precise any specific gene responsible for the syndrome. Only analysis of large cohorts of MRKH patients will certainly help to delineate new candidate genes and to establish phenotype/genotype correlations necessary for the genetic diagnosis of the syndrome.

## Diagnostic methods

### Transabdominal ultrasonography

Transabdominal ultrasonograph is a simple and noninvasive method, and must be the first investigation in evaluating patients with suspected Müllerian aplasia. This technique reveals an absence of the uterine structure between the bladder and the rectum. However, a quadrangular retro-vesical structure may be wrongly identified as a hypoplasic or juvenile uterus: this fact corresponds to the vestigial lamina located underneath the peritoneal fold, itself situated transversally to the posterior side of the bladder, where uterosacral ligaments attach. Since the vestigial lamina shows no cavity, there is no evidence of a hyperechogenic line, which normally corresponds to the uterine mucous membrane [91]. Finally, renal malformations must be systematically evaluated during this scan.

### Magnetic resonance imaging (MRI)

MRI is a non-invasive technique that provides a more sensitive and more specific means of diagnosis than ultrasonography. It should be performed when ultrasonographic findings are inconclusive or incomplete, since failure to clearly identify the uterus or Müllerian rudiments or ovaries does not necessarily imply their absence. MRI allows an accurate evaluation of the uterine aplasia, as well as a clear visualization of the rudimentary horns and ovaries [92,93]. The uterine aplasia is best characterized on sagittal images, while vaginal aplasia is best evidenced on transverse images [94]. Moreover, MRI can be used at the same time to search for associated renal and skeletal malformations.

### Celioscopy

This is an invasive technique requiring hospitalization and anesthesia. It is performed in cases of doubtful diagnosis after ultrasonography and/or MRI. Celioscopy is nowadays mainly reserved for women in whom interventional therapy is likely to be undertaken (construction of a neo-vagina: see Treatment section). It defines the precise anatomical location and abnormalities of the uterus, the possible tubar remnants, the vestigial lamina and the ovaries.

### Biological status

The karyotype of MRKH patients is always 46, XX with no visible chromosome modification. The endocrine balance (plasmatic follicle stimulating hormone (FSH), luteinizing hormone (LH) and 17**ß**-oestradiol) is normal and provides evidence of normal and functional ovaries [15,95]. There is no external or endocrine sign of hyperandrogenism, as shown by a normal plasmatic level of testosterone, delta-4-androstenedione, 17-hydroxyprogesterone and dehydroepiandrosterone.

Once MRKH syndrome is diagnosed, a full check-up must be undertaken to search for associated malformations. Since renal and skeletal abnormalities may not be symptomatic, it is necessary to perform at least transabdominal ultrasonography and spine radiography. In case of suspicion of hearing impairment and/or a cardiac anomaly, complementary audiogram and/or heart echography must also be carried out.

Moreover, when diagnosing an MRKH syndrome in a patient, it is important to consider the family history. Depending on the background, investigation of the patient's relatives may also be recommended, mainly for renal but also for skeletal malformations.

## Differential diagnosis

Differential diagnosis of Müllerian aplasia includes patients presenting with primary amenorrhea and with normal secondary sexual characteristics (Table 1). This should first lead to exclusion of gonadal dysgenesis. The differential diagnosis includes congenital absence of uterus and vagina (aplasia or agenesis), isolated vaginal atresia and androgen insensitivity [96,97]. Transverse vaginal septum and imperforate hymen, which can be initially misleading, are not included. Indeed, patients with these latter conditions have normal cervix and uterus, both of which are palpable on rectal examination. Ultrasonography can be used to define the Müllerian structures in infrequent cases where palpation is unrevealing.

**Table 1:** Summary of differential diagnosis between MRKH syndrome and isolated vaginal atresia, WNT4 syndrome, and androgen insensitivity syndrome.

	**MRKH/MURCS**	**Isolated vaginal atresia**	**WNT4 syndrome**	**Androgen insensitivity**
**Upper vagina**	Absent	Variable	Absent	Absent
**Uterus**	Absent	Present	Absent	Absent
**Gonads**	Ovary	Ovary	Masculinized ovary	Testis
**Breast development**	Normal	Normal	Normal	Normal
**Pubic-hair development**	Normal	Normal	Normal	Sparse
**Hyperandrogenism**	No	No	Yes	No
**Karyotype**	46, XX	46, XX	46, XX	46, XY

### Isolated vaginal atresia

Questioning will generally reveal pelvic pain in association with cryptomenorrhea on physical examination. Vaginal atresia is found in various syndromes, mainly Winter syndrome (characterized by renal, genital, and middle ear anomalies) (OMIM 267400) [98,99], and McKusick-Kaufman syndrome, which associates hydrometrocolpos, postaxial polydactyly and congenital heart malformation (OMIM 236700) and is due to mutations in the *MKKS *gene located on chromosome 20p12 [100]. It is noteworthy that while partial or total Müllerian aplasia found in MRKH syndrome confers irreversible sterility, vaginal atresia can be surgically corrected to permit pregnancy [99].

### WNT4 defects

To date, only two cases of WNT4 defects have been published [80,81]. This condition is similar but distinct from MRKH syndrome (see Etiology section) and may therefore lead to confusion. It seems quite clear that other cases will soon be reported in the literature, making it important to include this new syndrome in the differential diagnosis of MRKH/MURCS. Evidence of hyperandrogenism in women presenting with normal female phenotype should then initially direct the clinicians to suspect WNT4 as a cause.

### Androgen insensitivity syndrome (AIS)

AIS, also called testicular feminization syndrome (TFM), (OMIM 300068), is a male pseuhermaphroditism disorder caused by mutations in the gene for the androgen receptor [101]. AIS is an X-linked recessive disorder in which affected males have female external genitalia, female breast development, blind vagina, absent uterus and female adnexa, and abdominal or inguinal testes. Partial androgen insensitivity results in hypospadias and micropenis with gynecomastia, thus the syndrome cannot be confused with MRKH syndrome.

### Müllerian derivative aplasia

Müllerian derivative aplasia, which may be suggestive of MRKH syndrome, has been described in association with gonadal dysgenesis. In this case, patients showed abnormal karyotypes, always involving the X chromosome, such as mosaicisms 45, X/46, X, dic(X) [27], 46, XX/45, X0 [102], 46, XX/47, XXX [103] or rearrangements/deletions such as 46, X, del(X)(pter-q22) [104], 46, X, i(Xq) [105] or more complex karyotypes [12]. However, MRKH syndrome does not seem to be an X-linked trait and it therefore appears that the X chromosome carries one or several genes involved in very early differentiation of at least both gonads and Müllerian ducts.

## Management including treatment

Young women diagnosed with MRKH syndrome suffer from extreme anxiety and very high psychological distress when they are told they have no uterus and vagina. Thus, it is recommended that the patient and family attend counseling before and throughout treatment. Group programs [106] and/or MRKH patients associations are also of great help. Indeed, psychological adjustment as well as medical attitude will be of great consequence for future decisions of creation of a neo-vagina and management of sterility [2,70,106].

### Treatment of utero-vaginal aplasia

Treatment consisting of creating a neovagina must be offered to patients only when they are ready to start sexual activity and also when they are emotionally mature. Treatment may be either surgical or nonsurgical, but the chosen method needs to be tailored to the individual needs, motivation of the patient and the options available [2,70,96,107]. There are two main types of procedure. The first one consists of the creation of a new cavity and can be nonsurgical or surgical. The second is vaginal replacement with a pre-existing canal lined with a mucous membrane (a segment of bowel).

#### Nonsurgical creation of a neovagina

The most commonly used nonsurgical procedure is Franck's dilator method. It involves the application, first by the clinician and then by the patient herself, of vaginal dilators (Hegar candles), progressively increasing in length and diameter. Dilators are placed on the perineal dimple for at least 20 minutes a day. A variation of this procedure, using a bicycle stool, was described by Ingram [108]. The whole process takes between six weeks and several months, with a success rate varying from 78% [109] to 92% [110]. Complications are rare; they generally consist of urethritis, cystitis, vesico- or retro-vaginal fistula and secondary prolapse. As this nonoperative approach is noninvasive and often successful, it is recommended as a first-line therapy. However, it can be applied only when the vaginal dimple is deep enough (2–4 cm).

#### Surgical creation of a neovagina

A number of techniques are appropriate for the correction of vaginal agenesis and there is no consensus regarding the best option, the approach being most often based on the surgeon's experience. Three methods are currently in use:

- The Abbe-McIndoe operation: this involves the dissection of a space between the rectum and the bladder, placement of a mold covered with a skin graft into the space, and diligent postoperative vaginal dilatation. Modifications of this procedure rely on spontaneous epithelialization or on the use of different materials such as peritoneum [111], minora labia grafting, or synthetic materials [112,113].

- The Vecchietti operation is a mixture of surgical and nonsurgical methods. It has been performed frequently in Europe over the last 20 years [70]. This procedure involves the creation of a neovagina *via *dilatation with a traction device attached to the abdomen, sutures placed subperitoneally by laparotomy, and a plastic olive placed in the vaginal dimple. A laparoscopic or celioscopic modification is often preferred and leads to comparable results [114].

- Sigmoidal colpoplasty: this technique involves vaginal replacement or creation of a neovagina by grafting a 12–18 cm long segment of sigmoid [115], providing that a single and/or left pelvic kidney does not impair the procedure. Sigmoidal colpoplasty is believed to be an efficient procedure giving excellent results, although complete adequacy for coital function often requires prolonged care and support [116].

In conclusion, nonsurgical creation of a neovagina should be the first-line approach, if suitable. When a surgical approach is chosen, the surgeon must be experienced with the procedure. Clinical follow-up and also regular intercourse take place in the mid- and long-term successful process. Above all, a careful psychological preparation of the patient before any treatment or intervention is of major importance.

Ultimately, infertility will be the most difficult aspect of the disorder for the patient to accept. Nowadays medical technologies allow, in many countries, women to appeal for *in vitro *fertilization of their own eggs and to use surrogate pregnancy [96]. However, the risk for transmission of the disease cannot be accurately evaluated, since very little is currently known about genetics of the MRKH syndrome. This strengthens the need for more research in the field.

## Unresolved questions

Can an equivalent MRKH syndrome or MURCS manifest in the male? Striking similarities found in male patients have raised the question [117,118]. Combinations of Wolffian duct agenesis or severe hypoplasia with or without renal and/or skeletal anomalies and/or hearing impairment have been described and include congenital unilateral renal agenesis associated with ipsilateral agenesis of the vas deferens [9,119,120], primary infertility due to azoospermia associated with Klippel-Feil anomaly [117], and segmentation abnormalities of the cervicothoracic spine and hearing impairment [121,122]. Interestingly, such male cases were found in families with female patients with MRKH syndrome [9]. It is noteworthy that in azoospermic patients, the infertility seems to be attributable to uni- or bilateral defects of vas deferens development, ranging from hypoplasia [117] to agenesis [120,122] and leading to a so-called obstructive azoospermia.

Since the designation MURCS association cannot apply to males, it was suggested that the male counterpart ARCS (Azoospermia, Renal anomalies, Cervicothoracic Spine dysplasia) would be a more suitable designation for this condition in males [121,122]. The acronym GRES (Genital Renal Ear Skeletal), which applies to both sexes [10], would be even more appropriate, especially when MURCS and ARCS are found together in the same family [9].
